# Creating a literature database of low-calorie sweeteners and health studies: evidence mapping

**DOI:** 10.1186/s12874-015-0105-z

**Published:** 2016-01-05

**Authors:** Ding Ding Wang, Marissa Shams-White, Oliver John M. Bright, J. Scott Parrott, Mei Chung

**Affiliations:** Department of Public Health and Community Medicine, Tufts University School of Medicine, Boston, MA USA; Department of Interdisciplinary Studies, Rutgers University School of Health Related Professions, Newark, NJ USA

**Keywords:** Evidence map, Evidence-based methodology, Low-calorie sweeteners, Artificial sweeteners, High intensity sweeteners

## Abstract

**Background:**

Evidence mapping is an emerging tool used to systematically identify, organize and summarize the quantity and focus of scientific evidence on a broad topic, but there are currently no methodological standards. Using the topic of low-calorie sweeteners (LCS) and selected health outcomes, we describe the process of creating an evidence-map database and demonstrate several example descriptive analyses using this database.

**Methods:**

The process of creating an evidence-map database is described in detail. The steps include: developing a comprehensive literature search strategy, establishing study eligibility criteria and a systematic study selection process, extracting data, developing outcome groups with input from expert stakeholders and tabulating data using descriptive analyses. The database was uploaded onto SRDR™ (Systematic Review Data Repository), an open public data repository.

**Results:**

Our final LCS evidence-map database included 225 studies, of which 208 were interventional studies and 17 were cohort studies. An example bubble plot was produced to display the evidence-map data and visualize research gaps according to four parameters: comparison types, population baseline health status, outcome groups, and study sample size. This plot indicated a lack of studies assessing appetite and dietary intake related outcomes using LCS with a sugar intake comparison in people with diabetes.

**Conclusion:**

Evidence mapping is an important tool for the contextualization of in-depth systematic reviews within broader literature and identifies gaps in the evidence base, which can be used to inform future research. An open evidence-map database has the potential to promote knowledge translation from nutrition science to policy.

**Electronic supplementary material:**

The online version of this article (doi:10.1186/s12874-015-0105-z) contains supplementary material, which is available to authorized users.

## Background

Evidence mapping is an emerging tool to systematically and comprehensively identify, organize and summarize the distribution of scientific evidence on a broad topic [1, 2]. It can be thought of as a first step in conducting a broad systematic review, or organizing several related systematic reviews of published literature. However, unlike a systematic review, evidence mapping does not require a risk-of-bias appraisal of the included studies or detailed extraction and synthesis of the studies’ findings [1, 3]. Instead, it descriptively summarizes the characteristics of existing literature, typically in tabular forms, known as evidence (gap) maps [4]. These descriptive analyses can clarify where there is sufficient evidence to inform policy development, as well as identify research-dense areas where systematic reviews can be pursued [1, 3–7]. Conversely, evidence maps can also clarify areas where there is missing or inadequate evidence, known as research gaps [8]. These gaps can inform researchers and policy makers where strategic research prioritization is needed for future, focused studies [1–6, 8–10]. Aside from these utilities, there are other versions of evidence mapping with different focuses. For example, a scoping map or scoping review emphasizes capturing the entire scope of the evidence and tends to include ongoing research [7]. The use of evidence mapping to present characteristics of relevant studies as well as research gaps can potentially provide a cost-effective methodology to facilitate evidence-based decision-making [2, 3, 6, 11].

There are currently no methodological standards for evidence mapping [12]. As described earlier, discrepancies exist between the terminology and methods used within evidence mapping. Various terms, including *evidence map*, *scoping review* and *systematic map*, are widely used within evidence mapping, yet there are some differences between them [7]. Nonetheless, the following key steps are generally followed in all types of evidence mapping: 1) Identifying a broad research area of interest and defining the key variables and framework for descriptive analysis; 2) Developing a thorough, clear and reproducible literature search strategy; 3) Establishing the a priori inclusion and exclusion criteria; and 4) Systematically extracting, coding, sorting and reporting the findings in a tabular evidence map. It is preferable to consult with key stakeholders to guide these steps and to validate the findings [2, 4, 13].

In this paper, we describe the process and methodology of creating an evidence map database, using the topic of low-calorie sweeteners (LCS) and selected health outcomes of interest as a worked example. We also demonstrate several example descriptive analyses by using data in the LCS evidence map to help identify potential gaps and future research directions.

## Methods

Seven steps were used to construct a LCS evidence-map database: 1) Identify the Scope of the Evidence Map; 2) Define the Roles and Responsibilities of Different Parties: Stakeholder Panel and Research Team; 3) Develop a Comprehensive Search Strategy; 4) Establish Study Eligibility Criteria and a Systematic Study Selection Process; 5) Carry Out Abstract Screening and Selection; 6) Carry Out Data Extraction; and 7) Classify Outcome Categories. Details are described as follows.

### Identify the scope of the evidence map

The initial scope of work was developed by the sponsor of this project in a request-for- proposals. The following five areas of research regarding the potential health effects of LCS were all considered and included: 1) energy sensing by the brain; 2) gut hormones that may influence energy homeostasis; 3) satiety and preference for taste; 4) eating behavior; and 5) body weight and composition. It is important to note that, aside from their initial feedback on our funded research proposal, the sponsor was not involved in the later refinement of the work scope or the process of creating the evidence-map database other than providing a list of selective citations for cross-referencing purposes.

### Establish roles and responsibilities of different parties: stakeholder panel and research team

A diverse stakeholder panel was recruited across eight areas of expertise: one physician, two dietitians, three policymakers, a research funder, academic researchers from five different fields, two representatives from the food industry, one journalist and one lay person. The panel served as a steering committee to guide the research team through the process of building the LCS evidence-map database. Specifically, the stakeholder panel helped refine the search strategy, finalize study eligibility criteria and define outcome groups for evidence map analysis and charting. Data screening, collection and analyses were performed by the research team. The research team included a methodologist with expertise in conducting and leading systematic reviews and meta-analyses, two team members with graduate-level epidemiology training and a research coordinator.

### Develop a comprehensive search strategy

The research team collected key search terms from previous reviews and systematic reviews on relevant topics to formulate the initial search strategy [14–16]. The search strategy was developed using both keywords and the National Library of Medicine’s Medical Subject Headings (MeSH). Duplicated citations were removed in MEDLINE® and Endnote®. The search was also cross-referenced with published systematic reviews to make sure all of the relevant articles were included. Publication citations were exported from electronic search interfaces and stored in a Microsoft Excel spreadsheet. The relevance of the publications was determined based on pre-determined inclusion and exclusion criteria using a two-step screening process: 1) title and abstract screening and 2) full-text article screening, which is described later in detail. The initial literature search and abstract screening were conducted by the research team. After the screening was completed, the panel provided input and suggested additional key terms to refine the search strategy. The research team then carried out this supplemental search, retrieved the citations and screened this additional round of abstracts. Included abstracts from both screening rounds were then merged into the same pool for full-text screening. The final search strategy of the LCS evidence map is described in Additional file 1: Table S1.

### Establish study eligibility criteria and a systematic study selection process

The research team used an iterative process to establish study eligibility criteria, with input from the stakeholder panel collected via webinars and online surveys. After 2 iterations, the stakeholders and research team decided that for studies to be included, they needed to be: 1) randomized or non-randomized, controlled, clinical trials or prospective cohort study designs, which are considered the strongest study designs for causal inferences; 2) investigating orally administered, FDA-approved LCS or LCS that are generally recognized as safe (GRAS); 3) reporting at least one health outcome within the five categories in our scope of work; 4) an English publication; and 5) human subjects research. Articles with study populations including adults, children, adolescents, pregnant women and infants greater than 6 months old were included, whereas studies in cancer patients and cancer outcomes were excluded because the project scope includes only FDA-approved LCS that have passed FDA’s toxicological safety assessment as food additives, which includes assessment for cancer risks. A search of systematic reviews and meta-analyses was also conducted in addition to the main search for reference-mining purposes.

### Carry out abstract screening and study selection

The titles, abstracts and keywords of all identified articles were screened using Abstrackr™, an open source, web-based, citation screening tool [17]. The research team screened study abstracts using inclusion and exclusion criteria and assigned a decision (“yes”, “maybe” or “no”) to each abstract. Two rounds of pilot tests for a total of 1000 abstracts were screened by all reviewers to calibrate screening accuracy between reviewers. Discrepancies of the pilot abstracts were discussed among reviewers until complete agreement was reached and team members fully understood the selection criteria. The remainder of the abstracts (*n* = 11,830) were single-screened due to the large number of abstracts and budgetary constraints. Abstracts with a “yes” or “maybe” label were both included in step 1 of the evidence map and the full-text articles of these abstracts were retrieved for full-text screening. Citations with a “no” label were saved for record. Because of the single screening process, it is possible that a few abstracts were mistakenly rejected during this screening phase. In an effort to ensure all key studies were included, all included citations were cross-referenced with the reference list of relevant systematic reviews and meta-analyses. In step 2 (full-text screening), we followed the predefined study eligibility criteria (Table 1) and recorded the reasons for rejection of each rejected article. About 5 % (*n* = 571) of the original citations were screened in for full-text review. Each full-text article was screened by one reviewer, and the screening decision of the first reviewer was then confirmed or disputed by a second reviewer. Discrepancies were resolved through consensus among all research team members.

**Table 1:** Study eligibility criteria

Inclusion criteria	Exclusion criteria
• English language • Human subjects • Interventions studies (randomized or non-randomized control trials or single) • Adults; Pregnant women and infants (>6 months) • Prospective cohort studies • FDA-approved sweeteners	• Animal studies • In vitro cell studies • Case–control, cross-sectional studies; Reviews, interviews, bibliographies, letters, or guidelines • Systematic reviews and meta-analysis • Cancer patients • Non-oral intake

### Carry out data extraction

The research team extracted data on a customized extraction form shared via Google Drive to facilitate collaboration among research team members and allow for simultaneous data entry. The minimal data to collect for an evidence-map database are the information related to the PI(E)COS (Population, Intervention/Exposure, Comparator, Outcome and Study design). The extraction form for the LCS evidence-map database was designed to collect the following data from each study: study design characteristics (study design, study duration), study population characteristics (baseline health status, age, sample size, anthropometrics), study interventions/exposures and comparisons (type of LCS, comparison or control group, number of people analyzed, form of administration), outcome information (all outcomes or endpoints of interest), the aim or hypothesis of the study and funding source of the study. The extraction form was designed and finalized by the research team lead, who has expertise in evidence-based research and methods. The evidence-map database was uploaded onto SRDR™, an open public data repository, and a database codebook is included in the supplemental material (Additional file 2) [18].

### Classifications of outcome categories

One of the most important features of an evidence map is the cataloging of the large number and variety of outcomes reported in the published literature. This step typically occurs after data extraction since the scope of an evidence-map database is large. Thus, it is often difficult to pre-define all outcome categories of interest. The research team worked with the stakeholder panel to classify outcomes into clinically and biologically meaningful outcome categories that could be used in evidence-map analyses. The research team recorded outcomes reported in each publication and took the first attempt in identifying clinically and biologically relevant groups. Standardized coding was then developed for each outcome category. Feedback was sought from the stakeholder panel, and the outcome categories and coding were modified based on the final consensus of the stakeholder panel. Table 2 shows the final list of outcomes for each outcome category that are reported in the studies included in the LCS evidence-map database. Specifically, outcomes related to appetite or satiety ratings such as hunger score and desire to eat were often rated by a visual analog scale (VAS) and were classified under the ‘Appetite’ category. Outcomes focused on neurological measurements and sensing signals by the brain were classified under the ‘Energy Sensing’ category. Body weight, body composition and changes in weight-related outcomes were classified under the ‘Body Weight or Composition’ category. The ‘Dietary Intake’ category included outcomes such as energy intake, dietary intake, food intake and carbohydrate intake, and finally the ‘Glycemic’ category included glucose, insulin and gastric hormones. Our stakeholder panel did not identify additional outcomes that were not reported in the literature. Both outcome categories and full outcome lists were included in the evidence-map database, which can be used in future analyses with current or new outcome category coding.

**Table 2:** Outcomes of interest by outcome groups in the LCS evidence-map database

Outcome groups	Outcomes of interest
Appetite	Appetite ratings using a visual analog scale (VAS), hunger, desire to eat, fullness, prospective consumption, thirst, motivational and behavioral factors reported through questionnaire
Energy sensing by brain	Neurological measurements (fMRI, EEG), sensory rating (sweetness, intensity, pleasantness, sensory specific satiation), taste, perception and preference, taste reaction time
Body weight or body composition	Body weight, body composition, BMI, waist circumferences, weight or BMI changes
Dietary intake	Energy intake, dietary intake, food intake, carbohydrate intake, sugar intake, salt intake, water intake
Glycemic	Glucose, Hemoglobin A1c (HbA1c), insulin concentration, insulin sensitivity, hypoglycemia, glucagon, glucose-dependent insulinotropic peptide (GIP), glucagon-like peptide-1 (GLP-1), peptide tyrosine tyrosine (PYY), cholecystokinin (CCK), enterostatin, ghrelin, leptin, somatostatin, oxyntomodulin

## Results

The following sections demonstrate several example descriptive analyses by using data in the LCS evidence map to help identify potential gaps and future research directions. Using data stored in the evidence-map database, descriptive analyses can be performed to describe study design and population characteristics. All analyses and charting were performed using Stata 2013 (Stata Statistical Software: Release 13. College Station, TX: StataCorp LP) and Microsoft Excel.

### Summarize study and sample characteristics

**Fig. 1:**
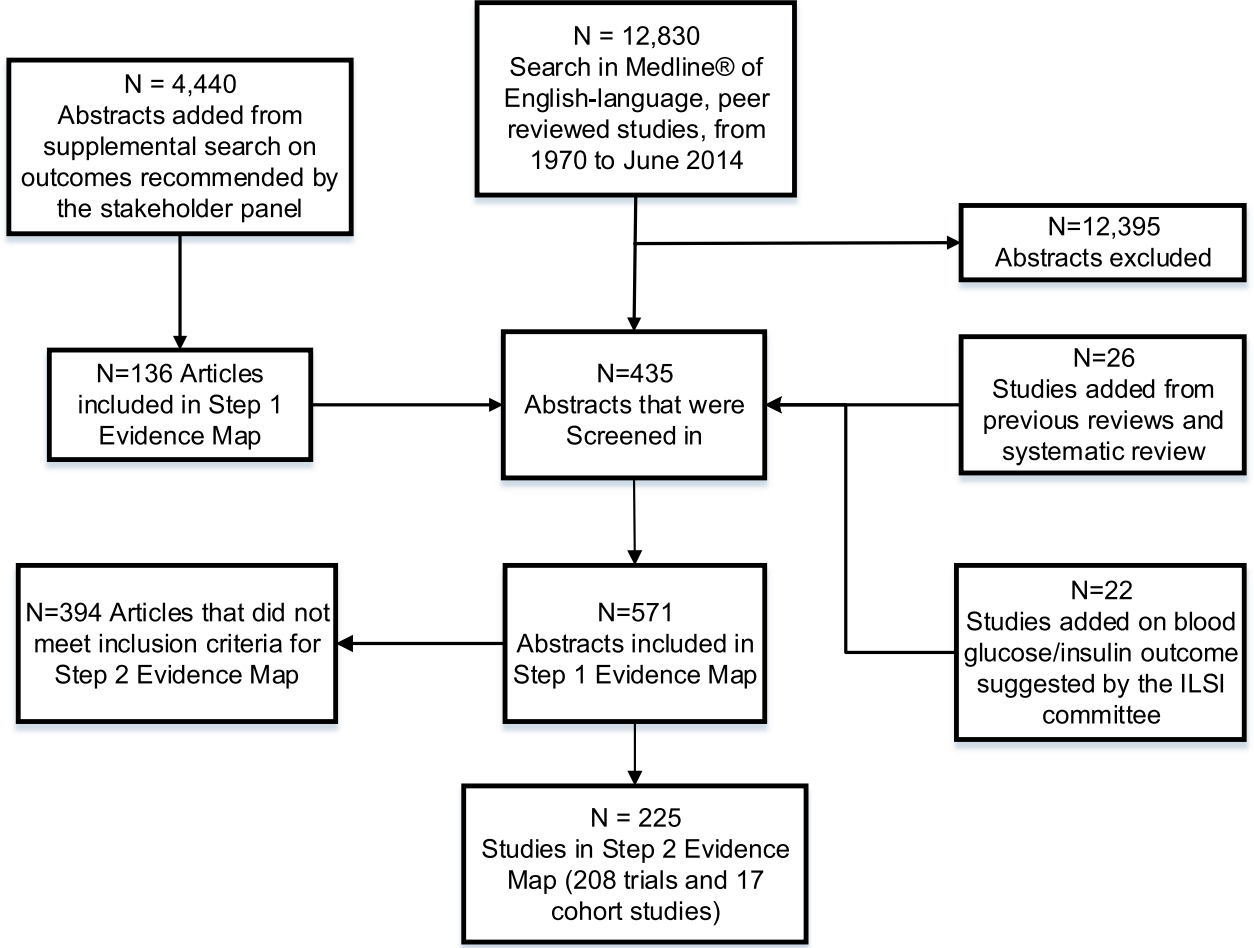
Literature search and selection process

The original search on MEDLINE® from inception to June 2014 yielded 12,830 citations. The supplemental search including terms recommended by the stakeholder panel yielded 4440 additional citations. In total, 17,270 relevant citations were screened. After title and abstract screening, 571 articles were included in full-text screening. Our final LCS evidence-map database included 225 studies, of which 208 were interventional studies and 17 were cohort studies. The literature search and study selection process are summarized in Fig. 1.

Among the 208 included intervention studies, 183 (88 %) were done in adults, 51 % of the study populations were male and 140 (68 %) of the studies included only healthy subjects with no known diseases. The mean sample size was 48 subjects (range: 2–732 subjects) and the mean BMI across studies was 25.6 kg/m^2^ (range: 16.1–42.3 kg/m^2^). The mean age was 31 years old (range: 8–73 years old). Fifty-seven percent of the trials were randomized cross-over trials and 28 % were randomized parallel controlled trials. Eleven percent of the trials were non-randomized crossover or parallel trials. More than half of the trials (60.1 %) were acute studies, defined as less than 1 day in study duration. Among the 17 cohort studies, the BMI (mean of the means) was 23.6 kg/m^2^ (range: 16.2–27.4 kg/m^2^). There were 6 studies conducted on children and 11 on adults. The mean age was 32 years old (range: 2–84 years old) with an average sample size of 15,430 subjects (164–31,940 subjects) (Table 3). Of the 17 cohort studies included, all were conducted in the United States and reported body weight and dietary intake outcomes. We did not identify any cohort studies that investigated appetite, glycemic or brain energy sensing outcomes. There were nine studies that used a food frequency questionnaire, five studies that used 24-h recalls or food diaries and three studies that used a specific beverage questionnaire. As for LCS intervention/exposure, eight studies reported it as LCS beverage intake, two as saccharin intake, and seven as unspecified, artificially-sweetened, carbonated drink intake, diet drink or diet soda intake.

**Table 3:** Summary of study design and population characteristics of the intervention in the evidence map

Intervention studies (*n* = 208)			
Study design	*N* (%)	Population	*N* (%)
RCT-c	119 (57.7)	Adults	183 (88 %)
RCT-p	58 (27.9)	Children	14 (7 %)
nRCT-c	21 (10.1)	Adolescents	2 (1 %)
nRCT-p	2 (1.0)	Mixed	6 (3 %)
Single arm	6 (2.9)	Unsure	2 (1 %)
Unclear	1 (0.5)	Baseline health	*N* (%)
Length	*N* (%)	Healthy	140 (68 %)
< 1 day	124 (60.1)	Overweight/Obese	19 (9 %)
2 day-1 month	37 (17.8)	Diabetic	17 (8 %)
1–6 months	30 (14.4)	Mixed	14 (7 %)
6 months–1 year	1 (0.5)	Other	17 (8 %)
> 1 year	7 (3.3)	Age, Mean (Range)	31 (8–73)
Not reported	8 (3.8)	BMI, Mean (Range)	25.6 (16.1–42.3)
Sample size, Mean (range)	48 (2–732)	% Male	51 % (0–100 %)

Data missing for age: *n* = 2, % male, *n* = 29, and BMI *n* = 87. *RCT*-*c* Randomized controlled trial-crossover design, *RCT*-*p* Randomized controlled trial-parallel design, *nRCT*-*c* Non-randomized controlled trial-crossover design, *nRCT*-*p* Non-randomized controlled trial-parallel design. Mixed or other: A mixture of healthy and overweight, healthy and diabetic, or overweight and diabetic population, and other population: patient or people with disease at baseline

### Summarize by types of outcomes

Studies in the evidence-map database can be summarized using the outcome categories described earlier. Among the 208 intervention studies identified, 84 studies reported appetite-related outcomes, 75 studies reported energy sensing-related outcomes, 35 studies reported body weight or body composition outcomes, 62 studies reported dietary intake-related outcomes and 76 studies reported glycemic outcomes. All 17 cohort studies reported body weight or body composition outcomes. Table 4 displays the type of LCS intervention or exposure, baseline health status of the study population, age of the study population, sample size and trial country by outcome category in the 208 intervention and 17 cohort studies. Briefly, the majority of the studies were conducted in the United States and in Europe using LCS in a beverage intervention among healthy, young adults.

**Table 4:** Study features of published LCS intervention studies (*n* = 208) and cohort studies (*n* = 17) by health outcome groups

Outcome group (number of studies)	Form of LCS intervention (number of studies)	Study design (number of studies)	Baseline health (number of studies)	Age group (mean)	Sample size (mean)	Country (number of studies)
Appetite (84)	Beverage (54), Food or meal (22), Supplement or oral rinse (8)	RCT-c (48), RCT-p (22), nRCT-c (11), nRCT-p (1), Single arm (1), unclear (1)	Healthy (66), Overweight/Obese (9), Mixed/other (9)	24.1	52	United States (41), Europe (31), Canada (9), Australia (3)
Alter energy sensing (75)	Beverage (58), Food or meal (10), Supplement or oral rinse (7)	RCT-c (48), RCT-p (10), nRCT-c (13), Single arm (3), unclear (1)	Healthy (54), Overweight/Obese (2), Diabetic (1), Mixed/other (18)	26.1	47	United States (43), Europe (23), Canada (7), Asia (2)
Body weight or body composition (35)	Beverage (14), Food or meal (13), Supplement or oral (8)	RCT-c (7), RCT-p (25), nRCT-c (2), nRCT-p (1)	Healthy (17), Overweight/Obese (8), Diabetic (5), Mixed/other (5)	35.6	92	United States (19), Europe (10), Asia (3), Africa (2), NR (1)
Body weight or body composition (17) cohort	Beverage (17)	Cohort (17)	Healthy (17)	32.0	15,430	United States (1), Europe (1)
Dietary Intake (62)	Beverage (39), Food or meal (19), Supplement or oral rinse (4)	RCT-c (33), RCT-p (20), nRCT-c (5), nRCT-p (2) Single arm (2)	Healthy (43), Overweight/Obese (12), Diabetic (1), Mixed/other (6)	27.2	39	United States (29), Europe (22), Canada (9), Australia (1), NR (1)
Glycemic (76)	Beverage (42), Food or meal (19), Supplement or oral rinse (14), Not reported (1)	RCT-c (51), RCT-p (21), nRCT-c (2), Single arm (2)	Healthy (39), Overweight/Obese (8), Diabetic (17), Mixed/other (12)	37.6	31	United States (28), Europe (27), Asia (8), Australia (7), Canada (3), Africa (1), Not reported (2)

*NR* Not reported, *RCT*-*c* Randomized controlled trial-crossover design, *RCT*-*p* Randomized controlled trial-parallel design, *nRCT*-*c* Non-randomized controlled trial-crossover design, *nRCT*-*p* Non-randomized controlled trial-parallel design

### Summarize publication patterns

**Fig. 2:**
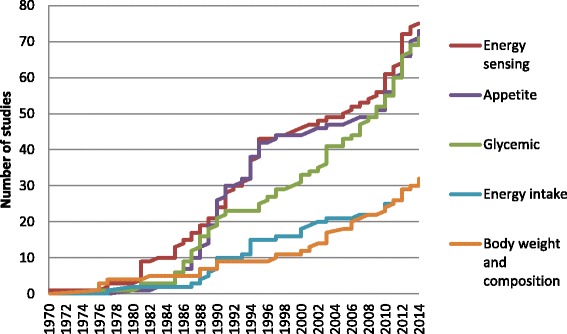
Cumulative growth of published LCS studies (*n* = 208)

A cumulative frequency chart of the number of publications by outcome categories was produced to show the cumulative publication growth over time. As shown in Fig. 2, there is an increasing trend in the number of publications reporting energy sensing and appetite-related outcomes from 1980 to 1995, and again from 2008 to 2014. The increasing interest of glycemic outcomes slowed down at 1991 and again spiked up at 2003. Up until 2014, there were larger cumulative numbers of publications reporting energy sensing, appetite and glycemic outcomes compared to energy intake and body weight or body composition outcomes (Fig. 2).

### Using a bubble plot to identify research gaps

A scatter plot is often used to identify relationships or patterns. A bubble plot is a variation of a weighted scatter plot, which was used here for the purpose of identifying research gaps. A bubble plot can graphically present multiple categorical data on study characteristics in a single, two-dimension chart by displaying the evidence-map data according to specific, special locations defined by the x-axis and y-axis, as well as according to the color, shape or size of bubbles. In the construction of the bubble plot using data in the LCS evidence-map database, the unit of the analyses was the individual study. One study could be included multiple times in an analysis because each study could report multiple outcomes of interest.

**Fig. 3:**
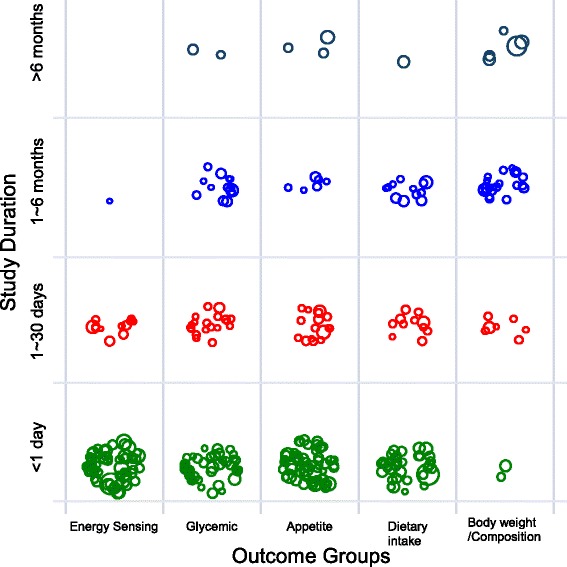
Example bubble plot of LCS studies by intervention duration and outcome categories. Legends: Each bubble represents one single study. Bubble size corresponds to study sample size

**Fig. 4:**
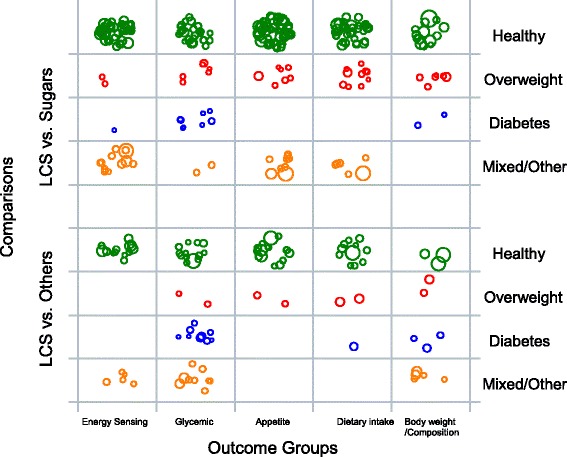
Example bubble plot of LCS studies by intervention type and health status. Legends: LCS vs. Sugars: An intervention comparison between LCS and sugars or sugars and LCS; LCS vs. Others: An intervention comparison between LCS and non-sugar arm or a single arm study. Other or mixed: A mixture of healthy and overweight, healthy and diabetic, or overweight and diabetic population, and other population: patient or people with disease at baseline. Each bubble represents one single study. Bubble size corresponds to study sample size

In the example bubble plots (Figs. 3 and 4), studies in the LCS evidence-map database are plotted into two-dimensional grids according to outcome categories and study characteristics such as intervention duration, intervention type, or population health status. Each data point is randomly scattered in each grid to maximize visualization of each bubble (so that not all bubbles overlaid with each other). The sample size of each study is shown as the weight of the bubble. Therefore, a larger bubble represents a larger study sample size. In the first example, bubble plot data points are grouped and plotted by study duration and outcome categories. This example bubble plot shows that the majority of LCS studies are acute (<1 day in duration) and short-term (1 to 30 days in duration) studies for all outcome categories, except for body weight or body composition outcomes. A majority of the studies report body weight or body composition outcomes with duration greater than 1 month (Fig. 3).

In the second example, bubble plot data points are grouped and plotted according to population health status and outcome categories. The plot is further stratified by two types of intervention and comparison designs: 1) LCS versus sugars (or vice versa) or 2) LCS versus other non-sugar comparisons (or vice versa) (Fig. 4). This example bubble plot shows that there are more studies in generally healthy populations compared to other population types across all outcome categories. The empty grids on the plot show that there is a lack of studies assessing appetite and dietary intake outcomes using a LCS intervention with a sugar intake comparison in people with diabetes. Most studies in people with diabetes reported body weight or body composition and glycemic outcomes. Among trials comparing LCS to sugar, there are a limited number of studies that investigated brain energy sensing outcomes in people who are overweight or who have diabetes.

It is important to note that the research gaps identified by the bubble plots do not necessarily equate to research needs. Besides the quantity and quality of the existing studies, the importance, desirability, feasibility, and potential impact of research gaps need to be considered to determine the research needs. For example, while a research gap exists regarding LCS vs. sugar intakes in diabetic populations, experts in this area would need to determine if there is a further need, given that individuals with diabetes need to limit their added sugars intake. The LCS evidence map database and analyses presented here were an integral component of a Future Research Needs (FRN) assessment project. The stakeholder panel in this project was assembled to provide input for the FRN project according to a framework for stakeholder engagement in patient-centered outcome research to identify research needs in the broad field of LCS and potential health-related outcomes [22].

## Discussion

**Fig. 5:**
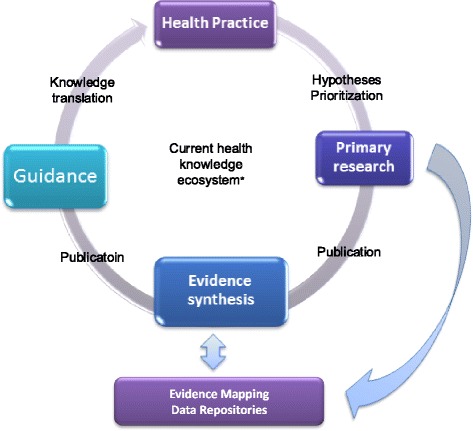
Role of evidence mapping in knowledge synthesis and translation*. *Modified from Elliott et al. Living systematic reviews: an emerging opportunity to narrow the evidence-practice gap. PLoS Med. 2014 Feb 18;11(2):e1001603

This paper reviewed the components of an evidence map and described the steps in the process of constructing a LCS evidence map as a worked example. The methodology presented to create the evidence map is replicable and helps readers understand the steps required to build a comprehensive database. These steps can be applied to create an evidence-map database for any research topic of interest. We have applied the same methodology to create two other evidence-map databases–one on the health effects of added sugars and another on fiber [19, 20]. The potential target users of evidence-map databases are researchers, research funders and policy makers, who can use the databases to identify research gaps and to evaluate the quantity of evidence accumulated for a specific research questions. Although evidence mapping is not a new method, it has only recently been applied in the fields of nutritional epidemiology and evidence-based nutrition. Because of the complexity of nutrition and chronic disease relationships, the decision makers often need to evaluate large arrays of interventions or exposures, comparisons and outcomes in comprehensive evidence reviews and syntheses. However, the resources (time and money) needed are increasing with the steadily upward trends in the number of scientific publications every year. Thus, establishing multiple, open evidence-map databases for nutrition research sets the much needed foundations for evidence-based nutrition guidelines or policy development. Open evidence-map databases increase transparency and can facilitate future updates. Evidence-map databases can be used to query what has been done based on a specific research question of interest in a broad area. Study characteristics can be summarized using the data in the evidence-map database to inform future study designs or to identify areas in need of more research with input from stakeholders. Conversely, evidence mapping can also help identify areas rich in studies, for which systematic reviews and meta-analyses can be conducted. Such systematic reviews and meta-analyses may be used in evaluating the science for public nutrition policy recommendations (e.g., Dietary Guidelines for Americans). By using a systematically collected and organized knowledge base, Research funders, researchers and practitioners can easily query and analyze the evidence-map databases to acquire information necessary for decision-making, such as directions for future research and the basis for evidence synthesis in a fraction of the time needed to conduct comprehensive literature reviews. Subsequently the process of knowledge translation from scientific findings into health practice or policy recommendations can be substantially shortened (Fig. 5).

Our LCS evidence-map database has several limitations. Due to limited resources, only the Medline® database was searched; thus the evidence-map database cannot be the sole source of data when used as the basis for a systematic review. We encourage future evidence map projects to include databases such as EMBASE, Cochrane, Scopus and other databases deemed appropriate to the research question. With more databases searched, it is expected that the number of abstracts and full-text articles to be screened will rise substantially. Hence, it is important to evaluate the project feasibility by looking at the number of search hits at the beginning of the project. Regardless of the comprehensive search strategy, it is likely we missed studies that did not include key LCS or outcome terms in the abstract or keywords. Our effort in addressing this limitation included cross-referencing studies cited from published LCS systematic reviews with our database, as well as conducting several supplemental searches to include additional key search terms. Furthermore, it is important to note again that evidence maps do not include quality (risk of bias) appraisal of the included studies. Thus, there can be a high volume of poor quality research and more research is still needed despite this high volume. There is a growing interest in engaging stakeholders in evidence synthesis processes for the potential benefits of improving relevant, enhancing quality, and increasing dissemination and uptake of evidence-based findings [21]. In this project, the iterative nature for defining outcomes of interest with input from the stakeholders inevitably created some subjectivity and uncertainties in the comprehensiveness of the search strategies because not all search terms were pre-defined according to the standards for conducting a systematic review. However, we could also argue that the stakeholders’ input helped strengthen a claim of comprehensiveness in capturing all important research areas of interest. Initially 12,830 citations were identified for further review, while using additional terms from the stakeholder panel, another 4400 citations were identified. This represents 34 % additional citations. Thus, the iterative and collaborative nature of the process is not a limitation, but rather strength for providing new dimensions to the subject. Future work should identify concrete criteria for evaluating the effectiveness of different methods, timing, and intensity of stakeholder engagement.

## Conclusion

Evidence mapping is a replicable evidence-based approach to identify, collect and evaluate the characteristics of published studies. The methodology is systematic and requires less time and effort than a systematic review to achieve an understanding and distribution of evidence. Evidence mapping can also be used to identify both research gaps, as well as areas of opportunity for systematic reviews. An open evidence-map database has the potential to promote knowledge translation from nutrition science to policy. To achieve this ultimate goal, continuous updating, data quality control and validation would be required.

## Additional files

Additional file 1: Table S1. LCS search strategy and supplemental search strategy. Supplemental material. Low-calorie sweetener database manual and codebook. (DOCX 75 kb) Additional file 2:
**LCS Database Manual and Codebook.** (DOCX 56 kb)

## Abbreviation

LCS: low-calorie-sweeteners
