# Supplementary material for: Creating a literature database of low-calorie sweeteners and health studies: evidence mapping
Source: BMC Med Res Methodol. 2016 Jan 5;16:1. doi: 10.1186/s12874-015-0105-z (PMC4700619; doi:10.1186/s12874-015-0105-z)
Supplement: Additional file 1: Table S1. — LCS search strategy and supplemental search strategy. Supplemental material. Low-calorie sweetener database manual and codebook. (DOCX 75 kb) [file 12874_2015_105_MOESM1_ESM.docx]

**Table S1. LCS Search Strategy and Supplemental Search Strategy.**

Database: Ovid MEDLINE(R) <1946 to May Week 1 2014>

Search Strategy:

--------------------------------------------------------------------------------

1 acesulfame K.mp. (114)

2 low calorie sweetener.mp. (44)

3 artificial sweetener$.mp. or exp Sweetening Agents/ (187234)

4 acesulfame potassium.mp. (25)

5 advantame.mp. (9)

6 alitame.mp. (32)

7 aspartame.mp. or exp Aspartame/ (1100)

8 cyclamate.mp. or exp Cyclamates/ (745)

9 D-tagatose.mp. (167)

10 erythritol.mp. or exp Erythritol/ (1856)

11 high intensity sweetener.mp. or exp Non-Nutritive Sweeteners/ (31)

12 high potency sweetener.mp. (12)

13 hydrogenated starch hydrolysate.mp. (9)

14 maltitol syrup.mp. (3)

15 sorbitol syrup.mp. or exp Sorbitol/ (14748)

16 intense sweetener.mp. (26)

17 isomaltulose.mp. (150)

18 isomalt.mp. (56)

19 lactitol.mp. (251)

20 Luo han guo extract.mp. (0)

21 cucurbitane glycosides.mp. (16)

22 mogroside.mp. (25)

23 maltitol.mp. (181)

24 mannitol.mp. or exp Mannitol/ (18554)

25 neotame.mp. (52)

26 nonnutritive sweetener.mp. (20)

27 polyol.mp. (2448)

28 rebaudioside A.mp. (83)

29 rebiana.mp. (4)

30 saccharin.mp. or exp Saccharin/ (4103)

31 sorbitol.mp. or exp Sorbitol/ (18757)

32 stevioside.mp. (256)

33 stevia extract.mp. (7)

34 stevia leaf extract.mp. (0)

35 sucralose.mp. (246)

36 trichlorogalactosucrose.mp. (3)

37 sugar alcohol.mp. or exp Sugar Alcohols/ (90930)

38 trehalose.mp. or exp Trehalose/ (5431)

39 xylitol.mp. or exp Xylitol/ (2736)

40 sweetening agent.mp. or exp Sweetening Agents/ (187217)

41 sugar substitute.mp. or exp Sweetening Agents/ (187230)

42 diet beverage.mp. (29)

43 diet drink.mp. (8)

44 diet soda.mp. (59)

45 diet soft drink.mp. (17)

46 low calorie sweetener$.mp. (44)

47 no sugar added.mp. (7)

48 non-caloric beverage.mp. (3)

49 non-caloric drink.mp. (1)

50 non-caloric soft drink.mp. (1)

51 reduced sugar.mp. (66)

52 sugar free.mp. (441)

53 Reduced calorie beverage.mp. (0)

54 reduced sugar beverage.mp. (0)

55 artificially sweetened.mp. (106)

56 or/1-55 (272974)

57 cephalic phase responses.mp. or exp Brain/ (977319)

58 immunoglobulins.mp. or exp Immunoglobulins/ (744664)

59 dorsal motor nucleus of the vagus.mp. (1187)

60 nucleus of the solitary tract.mp. or exp Solitary Nucleus/ (4301)

61 lateral hypothalamus.mp. (3187)

62 exp Ventromedial Hypothalamic Nucleus/ or ventromedial hypothalamus.mp. (2647)

63 exp Orexin Receptors/ or orexins.mp. (2696)

64 thyrotropin-releasing hormone.mp. or exp Thyrotropin-Releasing Hormone/ (13489)

65 exp Amygdala/ or Amygdala response.mp. (17360)

66 Blood oxygen level-dependent.mp. (2183)

67 exp Taste/ or Taste.mp. (27564)

68 Flavor.mp. (4802)

69 visual analog score.mp. (312)

70 Oral somatosensation.mp. (3)

71 Retronasal olfaction.mp. (28)

72 Taste perception.mp. or exp Taste Perception/ (1145)

73 taste physiology.mp. (66)

74 Choice behavior.mp. or exp Choice Behavior/ (39652)

75 Food habits.mp. or exp Food Habits/ (141502)

76 Food preferences.mp. or exp Food Preferences/ (10251)

77 Instinct.mp. or exp Instinct/ (1400)

78 Flavor processing.mp. (9)

79 Nucleus accumbens.mp. or exp Nucleus Accumbens/ (15736)

80 opioid.mp. or exp Analgesics, Opioid/ (122315)

81 ventral pallidum.mp. (891)

82 (limbic or mesolimbic).mp. [mp=title, abstract, original title, name of substance word, subject heading word, keyword heading word, protocol supplementary concept word, rare disease supplementary concept word, unique identifier] (24258)

83 exp Dopamine/ or dopamine.mp. (134094)

84 exp Leptin/ or leptin.mp. (23925)

85 brain mapping.mp. or exp Brain Mapping/ (70479)

86 magnetic resonance imaging.mp. or exp Magnetic Resonance Imaging/ (338919)

87 positron-emission tomography.mp. or exp Positron-Emission Tomography/ (48970)

88 or/57-87 (2365770)

89 56 and 88 (35217)

90 limit 89 to (addresses or bibliography or biography or comment or congresses or consensus development conference or consensus development conference, nih or dictionary or directory or duplicate publication or editorial or in vitro or interview or lectures or letter or news or newspaper article or "review") (5010)

91 89 not 90 (30207)

92 exp case control study/ (657418)

93 (case$ and control$).tw. (303774)

94 92 or 93 (875362)

95 exp Cross-Sectional Studies/ or cross-sectiona.ti,ab. or "prevalence study".ti,ab. (176278)

96 94 or 95 (1033041)

97 91 not 96 (28920)

**98 limit 97 to (english language and humans) (9909)**

99 acesulfame K.mp. (114)

100 low calorie sweetener.mp. (44)

101 artificial sweetener.mp. or exp Sweetening Agents/ (187234)

102 acesulfame potassium.mp. (25)

103 advantame.mp. (9)

104 alitame.mp. (32)

105 aspartame.mp. or exp Aspartame/ (1100)

106 cyclamate.mp. or exp Cyclamates/ (745)

107 D-tagatose.mp. (167)

108 erythritol.mp. or exp Erythritol/ (1856)

109 high intensity sweetener.mp. or exp Non-Nutritive Sweeteners/ (31)

110 high potency sweetener.mp. (12)

111 hydrogenated starch hydrolysate.mp. (9)

112 maltitol syrup.mp. (3)

113 sorbitol syrup.mp. or exp Sorbitol/ (14748)

114 intense sweetener.mp. (26)

115 isomaltulose.mp. (150)

116 isomalt.mp. (56)

117 lactitol.mp. (251)

118 Luo han guo extract.mp. (0)

119 cucurbitane glycosides.mp. (16)

120 mogroside.mp. (25)

121 maltitol.mp. (181)

122 mannitol.mp. or exp Mannitol/ (18554)

123 neotame.mp. (52)

124 nonnutritive sweetener.mp. (20)

125 polyol.mp. (2448)

126 rebaudioside A.mp. (83)

127 rebiana.mp. (4)

128 saccharin.mp. or exp Saccharin/ (4103)

129 sorbitol.mp. or exp Sorbitol/ (18757)

130 stevioside.mp. (256)

131 stevia extract.mp. (7)

132 stevia leaf extract.mp. (0)

133 sucralose.mp. (246)

134 trichlorogalactosucrose.mp. (3)

135 sugar alcohol.mp. or exp Sugar Alcohols/ (90930)

136 trehalose.mp. or exp Trehalose/ (5431)

137 xylitol.mp. or exp Xylitol/ (2736)

138 sweetening agent.mp. or exp Sweetening Agents/ (187217)

139 sugar substitute.mp. or exp Sweetening Agents/ (187230)

140 diet beverage.mp. (29)

141 diet drink.mp. (8)

142 diet soda.mp. (59)

143 diet soft drink.mp. (17)

144 low calorie sweetener.mp. (44)

145 no sugar added.mp. (7)

146 non-caloric beverage.mp. (3)

147 non-caloric drink.mp. (1)

148 non-caloric soft drink.mp. (1)

149 reduced sugar.mp. (66)

150 sugar free.mp. (441)

151 Reduced calorie beverage.mp. (0)

152 reduced sugar beverage.mp. (0)

153 artificially sweetened.mp. (106)

154 or/99-153 (272974)

155 limit 154 to (english language and humans) (87874)

156 Eating disorders.mp. or exp Eating Disorders/ (24271)

157 Analgesics, opioid.mp. or exp Analgesics, Opioid/ (91705)

158 Brain-derived neurotrophic factor.mp. or exp Brain-Derived Neurotrophic Factor/ (11787)

159 Cannabinoid receptor modulators.mp. or exp Cannabinoid Receptor Modulators/ (8933)

160 exp Ghrelin/ or Ghrelin.mp. (6374)

161 Reward.mp. or exp Reward/ (35776)

162 Impulsivitiy.mp. [mp=title, abstract, original title, name of substance word, subject heading word, keyword heading word, protocol supplementary concept word, rare disease supplementary concept word, unique identifier] (0)

163 exp Obsessive-Compulsive Disorder/ or Obsessive-compulsive.mp. (14310)

164 exp Bulimia/ or Bulimic.mp. (5520)

165 exp Impulsive Behavior/ or impulsive.mp. (16014)

166 or/156-165 (203038)

167 155 and 166 (965)

168 limit 167 to (addresses or bibliography or biography or comment or congresses or consensus development conference or consensus development conference, nih or dictionary or directory or duplicate publication or editorial or in vitro or interview or lectures or letter or news or newspaper article or "review") (243)

169 167 not 168 (722)

170 exp case control study/ (657418)

171 (case$ and control$).tw. (303774)

172 170 or 171 (875362)

173 exp Cross-Sectional Studies/ or cross-sectiona.ti,ab. or "prevalence study".ti,ab. (176278)

174 172 or 173 (1033041)

175 169 not 174 (665)

**176 limit 175 to (english language and humans) (665)**

177 acesulfame K.mp. (114)

178 low calorie sweetener.mp. (44)

179 artificial sweetener.mp. or exp Sweetening Agents/ (187234)

180 acesulfame potassium.mp. (25)

181 advantame.mp. (9)

182 alitame.mp. (32)

183 aspartame.mp. or exp Aspartame/ (1100)

184 cyclamate.mp. or exp Cyclamates/ (745)

185 D-tagatose.mp. (167)

186 erythritol.mp. or exp Erythritol/ (1856)

187 high intensity sweetener.mp. or exp Non-Nutritive Sweeteners/ (31)

188 high potency sweetener.mp. (12)

189 hydrogenated starch hydrolysate.mp. (9)

190 maltitol syrup.mp. (3)

191 sorbitol syrup.mp. or exp Sorbitol/ (14748)

192 intense sweetener.mp. (26)

193 isomaltulose.mp. (150)

194 isomalt.mp. (56)

195 lactitol.mp. (251)

196 Luo han guo extract.mp. (0)

197 cucurbitane glycosides.mp. (16)

198 mogroside.mp. (25)

199 maltitol.mp. (181)

200 mannitol.mp. or exp Mannitol/ (18554)

201 neotame.mp. (52)

202 nonnutritive sweetener.mp. (20)

203 polyol.mp. (2448)

204 rebaudioside A.mp. (83)

205 rebiana.mp. (4)

206 saccharin.mp. or exp Saccharin/ (4103)

207 sorbitol.mp. or exp Sorbitol/ (18757)

208 stevioside.mp. (256)

209 stevia extract.mp. (7)

210 stevia leaf extract.mp. (0)

211 sucralose.mp. (246)

212 trichlorogalactosucrose.mp. (3)

213 sugar alcohol.mp. or exp Sugar Alcohols/ (90930)

214 trehalose.mp. or exp Trehalose/ (5431)

215 xylitol.mp. or exp Xylitol/ (2736)

216 sweetening agent.mp. or exp Sweetening Agents/ (187217)

217 sugar substitute.mp. or exp Sweetening Agents/ (187230)

218 diet beverage.mp. (29)

219 diet drink.mp. (8)

220 diet soda.mp. (59)

221 diet soft drink.mp. (17)

222 low calorie sweetener.mp. (44)

223 no sugar added.mp. (7)

224 non-caloric beverage.mp. (3)

225 non-caloric drink.mp. (1)

226 non-caloric soft drink.mp. (1)

227 reduced sugar.mp. (66)

228 sugar free.mp. (441)

229 Reduced calorie beverage.mp. (0)

230 reduced sugar beverage.mp. (0)

231 artificially sweetened.mp. (106)

232 or/177-231 (272974)

233 limit 232 to (english language and humans) (87874)

234 Gustatory pathway.mp. (41)

235 energy homeostasis.mp. (4694)

236 fMRI.mp. or exp Magnetic Resonance Imaging/ (311835)

237 PET.mp. (51357)

238 Functional brain imaging.mp. or exp Functional Neuroimaging/ (71793)

239 Activation likelihood estimation.mp. (147)

240 exp Incretins/ or exp Gastric Inhibitory Polypeptide/ or exp Glucagon-Like Peptide 1/ or Incretin hormones.mp. (6467)

241 peptide YY.mp. or exp Peptide YY/ (2307)

242 cholecystokinin.mp. or exp Cholecystokinin/ (17271)

243 pancreatic polypeptide.mp. or exp Pancreatic Polypeptide/ (4007)

244 amylin.mp. (1586)

245 oxyntomodulin.mp. or exp Oxyntomodulin/ (256)

246 or/234-245 (432949)

247 233 and 246 (5144)

248 limit 247 to (addresses or bibliography or biography or comment or congresses or consensus development conference or consensus development conference, nih or dictionary or directory or duplicate publication or editorial or in vitro or interview or lectures or letter or news or newspaper article or "review") (689)

249 247 not 248 (4455)

250 exp case control study/ (657418)

251 (case$ and control$).tw. (303774)

252 250 or 251 (875362)

253 exp Cross-Sectional Studies/ or cross-sectiona.ti,ab. or "prevalence study".ti,ab. (176278)

254 252 or 253 (1033041)

255 249 not 254 (3818)

**256 limit 255 to (english language and humans) (3818)**

257 acesulfame K.mp. (114)

258 low calorie sweetener.mp. (44)

259 artificial sweetener.mp. or exp Sweetening Agents/ (187234)

260 acesulfame potassium.mp. (25)

261 advantame.mp. (9)

262 alitame.mp. (32)

263 aspartame.mp. or exp Aspartame/ (1100)

264 cyclamate.mp. or exp Cyclamates/ (745)

265 D-tagatose.mp. (167)

266 erythritol.mp. or exp Erythritol/ (1856)

267 high intensity sweetener.mp. or exp Non-Nutritive Sweeteners/ (31)

268 high potency sweetener.mp. (12)

269 hydrogenated starch hydrolysate.mp. (9)

270 maltitol syrup.mp. (3)

271 sorbitol syrup.mp. or exp Sorbitol/ (14748)

272 intense sweetener.mp. (26)

273 isomaltulose.mp. (150)

274 isomalt.mp. (56)

275 lactitol.mp. (251)

276 Luo han guo extract.mp. (0)

277 cucurbitane glycosides.mp. (16)

278 mogroside.mp. (25)

279 maltitol.mp. (181)

280 mannitol.mp. or exp Mannitol/ (18554)

281 neotame.mp. (52)

282 nonnutritive sweetener.mp. (20)

283 polyol.mp. (2448)

284 rebaudioside A.mp. (83)

285 rebiana.mp. (4)

286 saccharin.mp. or exp Saccharin/ (4103)

287 sorbitol.mp. or exp Sorbitol/ (18757)

288 stevioside.mp. (256)

289 stevia extract.mp. (7)

290 stevia leaf extract.mp. (0)

291 sucralose.mp. (246)

292 trichlorogalactosucrose.mp. (3)

293 sugar alcohol.mp. or exp Sugar Alcohols/ (90930)

294 trehalose.mp. or exp Trehalose/ (5431)

295 xylitol.mp. or exp Xylitol/ (2736)

296 sweetening agent.mp. or exp Sweetening Agents/ (187217)

297 sugar substitute.mp. or exp Sweetening Agents/ (187230)

298 diet beverage.mp. (29)

299 diet drink.mp. (8)

300 diet soda.mp. (59)

301 diet soft drink.mp. (17)

302 low calorie sweetener.mp. (44)

303 no sugar added.mp. (7)

304 non-caloric beverage.mp. (3)

305 non-caloric drink.mp. (1)

306 non-caloric soft drink.mp. (1)

307 reduced sugar.mp. (66)

308 sugar free.mp. (441)

309 Reduced calorie beverage.mp. (0)

310 reduced sugar beverage.mp. (0)

311 artificially sweetened.mp. (106)

312 or/257-311 (272974)

313 limit 312 to (english language and humans) (87874)

314 exp Satiation/ (4532)

315 exp Satiety Response/ (2087)

316 (appetite adj2 alteration$).mp. (70)

317 exp Appetite Regulation/ (2363)

318 ("intake regulation$" adj2 food$).mp. (263)

319 (satiety-related adj2 hormone$).mp. (13)

320 ((satiety adj2 hormone$) or (hunger adj2 hormone$)).mp. (274)

321 addictive behavior$.mp. or exp addictive behavior/ (6280)

322 uncontrollable eating$.mp. or exp Obesity, Morbid/ or exp Eating Disorders/ or exp Bulimia/ (35556)

323 abusive eating$.mp. or exp Eating Disorders/ (22123)

324 excessive craving$.mp. or exp Behavior, Addictive/ (5511)

325 binge eating$.mp. or exp Bulimia/ (6624)

326 exp Energy Intake/ (34485)

327 (caloric adj2 intake$).mp. (5064)

328 exp Ghrelin/ (5116)

329 exp Leptin/ (17840)

330 ("total peptide tyrosine" or "total peptide tyrosine-tyrosine" or "tyrosine" or "PYY").mp. (173994)

331 ("total glucagon-like peptide-1" or GLP-1 or GLP1 or "glucagon like peptide 1").mp. [mp=title, abstract, original title, name of substance word, subject heading word, keyword heading word, protocol supplementary concept word, rare disease supplementary concept word, unique identifier] (6835)

332 or/314-331 (281127)

333 313 and 332 (3865)

334 limit 333 to (addresses or bibliography or biography or comment or congresses or consensus development conference or consensus development conference, nih or dictionary or directory or duplicate publication or editorial or in vitro or interview or lectures or letter or news or newspaper article or "review") (797)

335 333 not 334 (3068)

336 exp case control study/ (657418)

337 (case$ and control$).tw. (303774)

338 336 or 337 (875362)

339 exp Cross-Sectional Studies/ or cross-sectiona.ti,ab. or "prevalence study".ti,ab. (176278)

340 338 or 339 (1033041)

341 335 not 340 (2833)

**342 limit 341 to (english language and humans) (2833)**

343 acesulfame K.mp. (114)

344 low calorie sweetener.mp. (44)

345 artificial sweetener.mp. or exp Sweetening Agents/ (187234)

346 acesulfame potassium.mp. (25)

347 advantame.mp. (9)

348 alitame.mp. (32)

349 aspartame.mp. or exp Aspartame/ (1100)

350 cyclamate.mp. or exp Cyclamates/ (745)

351 D-tagatose.mp. (167)

352 erythritol.mp. or exp Erythritol/ (1856)

353 high intensity sweetener.mp. or exp Non-Nutritive Sweeteners/ (31)

354 high potency sweetener.mp. (12)

355 hydrogenated starch hydrolysate.mp. (9)

356 maltitol syrup.mp. (3)

357 sorbitol syrup.mp. or exp Sorbitol/ (14748)

358 intense sweetener.mp. (26)

359 isomaltulose.mp. (150)

360 isomalt.mp. (56)

361 lactitol.mp. (251)

362 Luo han guo extract.mp. (0)

363 cucurbitane glycosides.mp. (16)

364 mogroside.mp. (25)

365 maltitol.mp. (181)

366 mannitol.mp. or exp Mannitol/ (18554)

367 neotame.mp. (52)

368 nonnutritive sweetener.mp. (20)

369 polyol.mp. (2448)

370 rebaudioside A.mp. (83)

371 rebiana.mp. (4)

372 saccharin.mp. or exp Saccharin/ (4103)

373 sorbitol.mp. or exp Sorbitol/ (18757)

374 stevioside.mp. (256)

375 stevia extract.mp. (7)

376 stevia leaf extract.mp. (0)

377 sucralose.mp. (246)

378 trichlorogalactosucrose.mp. (3)

379 sugar alcohol.mp. or exp Sugar Alcohols/ (90930)

380 trehalose.mp. or exp Trehalose/ (5431)

381 xylitol.mp. or exp Xylitol/ (2736)

382 sweetening agent.mp. or exp Sweetening Agents/ (187217)

383 sugar substitute.mp. or exp Sweetening Agents/ (187230)

384 diet beverage.mp. (29)

385 diet drink.mp. (8)

386 diet soda.mp. (59)

387 diet soft drink.mp. (17)

388 low calorie sweetener.mp. (44)

389 no sugar added.mp. (7)

390 non-caloric beverage.mp. (3)

391 non-caloric drink.mp. (1)

392 non-caloric soft drink.mp. (1)

393 reduced sugar.mp. (66)

394 sugar free.mp. (441)

395 Reduced calorie beverage.mp. (0)

396 reduced sugar beverage.mp. (0)

397 artificially sweetened.mp. (106)

398 or/343-397 (272974)

399 limit 398 to (english language and humans) (87874)

400 exp Weight Loss/ (28002)

401 exp Body Weight Changes/ (49112)

402 exp Body Weight/ (345744)

403 (reduction$ adj2 adiposity).mp. [mp=title, abstract, original title, name of substance word, subject heading word, keyword heading word, protocol supplementary concept word, rare disease supplementary concept word, unique identifier] (86)

404 exp Weight Gain/ (22503)

405 exp Obesity/ or exp Overweight/ (144221)

406 (obesit$ adj2 morbid).mp. [mp=title, abstract, original title, name of substance word, subject heading word, keyword heading word, protocol supplementary concept word, rare disease supplementary concept word, unique identifier] (12937)

407 exp Obesity Hypoventilation Syndrome/ (619)

408 (pickwickian adj2 syndrome$).mp. [mp=title, abstract, original title, name of substance word, subject heading word, keyword heading word, protocol supplementary concept word, rare disease supplementary concept word, unique identifier] (247)

409 exp Obesity, Abdominal/ (1404)

410 ((central adj2 obesit$) or (visceral adj2 obesit$)).mp. [mp=title, abstract, original title, name of substance word, subject heading word, keyword heading word, protocol supplementary concept word, rare disease supplementary concept word, unique identifier] (3873)

411 exp Body Constitution/ (472417)

412 (body weights and measures).mp. [mp=title, abstract, original title, name of substance word, subject heading word, keyword heading word, protocol supplementary concept word, rare disease supplementary concept word, unique identifier] (3802)

413 (body adj2 (weight$ or measure$)).mp. [mp=title, abstract, original title, name of substance word, subject heading word, keyword heading word, protocol supplementary concept word, rare disease supplementary concept word, unique identifier] (276566)

414 exp Body Fat Distribution/ (6789)

415 ("body fat" adj2 patterning).mp. [mp=title, abstract, original title, name of substance word, subject heading word, keyword heading word, protocol supplementary concept word, rare disease supplementary concept word, unique identifier] (37)

416 (fat adj2 patterning).mp. [mp=title, abstract, original title, name of substance word, subject heading word, keyword heading word, protocol supplementary concept word, rare disease supplementary concept word, unique identifier] (162)

417 exp Adiposity/ (5385)

418 exp Body Mass Index/ (79416)

419 (quetelet$ adj2 index).mp. [mp=title, abstract, original title, name of substance word, subject heading word, keyword heading word, protocol supplementary concept word, rare disease supplementary concept word, unique identifier] (686)

420 exp Body Size/ (362574)

421 exp Waist Circumference/ (4508)

422 exp Waist-Hip Ratio/ (2867)

423 exp Skinfold Thickness/ (5604)

424 or/400-423 (584691)

425 399 and 424 (6938)

426 limit 425 to (addresses or bibliography or biography or comment or congresses or consensus development conference or consensus development conference, nih or dictionary or directory or duplicate publication or editorial or in vitro or interview or lectures or letter or news or newspaper article or "review") (1362)

427 425 not 426 (5576)

428 exp case control study/ (657418)

429 (case$ and control$).tw. (303774)

430 428 or 429 (875362)

431 exp Cross-Sectional Studies/ or cross-sectiona.ti,ab. or "prevalence study".ti,ab. (176278)

432 430 or 431 (1033041)

433 427 not 432 (5059)

**434 limit 433 to (english language and humans and yr="2013") (258)**

435 98 or 176 or 256 or 342 or 434 **(12830)**

***************************

Supplemental Search Strategy

Database: Ovid MEDLINE(R) <1946 to September Week 4 2014>, Ovid MEDLINE(R) In-Process & Other Non-Indexed Citations <October 03, 2014>

Search Strategy:

----------------------------------------------------------------------

1 acesulfame K.mp. (129)

2 low calorie sweetener.mp. (47)

3 artificial sweetener.mp. or exp Sweetening Agents/ (191253)

4 acesulfame potassium.mp. (36)

5 advantame.mp. (10)

6 alitame.mp. (34)

7 aspartame.mp. or exp Aspartame/ (1204)

8 cyclamate.mp. or exp Cyclamates/ (775)

9 D-tagatose.mp. (196)

10 erythritol.mp. or exp Erythritol/ (1988)

11 high intensity sweetener.mp. or exp Non-Nutritive Sweeteners/ (42)

12 high potency sweetener.mp. (14)

13 hydrogenated starch hydrolysate.mp. (9)

14 maltitol syrup.mp. (3)

15 sorbitol syrup.mp. or exp Sorbitol/ (14965)

16 intense sweetener.mp. (28)

17 isomaltulose.mp. (162)

18 isomalt.mp. (68)

19 lactitol.mp. (269)

20 Luo han guo extract.mp. (0)

21 cucurbitane glycosides.mp. (19)

22 mogroside.mp. (29)

23 maltitol.mp. (201)

24 mannitol.mp. or exp Mannitol/ (19901)

25 neotame.mp. (57)

26 nonnutritive sweetener.mp. (22)

27 polyol.mp. (2891)

28 rebaudioside A.mp. (110)

29 rebiana.mp. (4)

30 saccharin.mp. or exp Saccharin/ (4360)

31 sorbitol.mp. or exp Sorbitol/ (19577)

32 stevioside.mp. (298)

33 stevia extract.mp. (12)

34 stevia leaf extract.mp. (0)

35 sucralose.mp. (312)

36 trichlorogalactosucrose.mp. (3)

37 sugar alcohol.mp. or exp Sugar Alcohols/ (92321)

38 trehalose.mp. or exp Trehalose/ (6034)

39 xylitol.mp. or exp Xylitol/ (3022)

40 sweetening agent.mp. or exp Sweetening Agents/ (191213)

41 sugar substitute.mp. or exp Sweetening Agents/ (191232)

42 diet beverage.mp. (31)

43 diet drink.mp. (9)

44 diet soda.mp. (74)

45 diet soft drink.mp. (19)

46 low calorie sweetener.mp. (47)

47 no sugar added.mp. (7)

48 non-caloric beverage.mp. (3)

49 non-caloric drink.mp. (2)

50 non-caloric soft drink.mp. (1)

51 reduced sugar.mp. (79)

52 sugar free.mp. (502)

53 Reduced calorie beverage.mp. (0)

54 reduced sugar beverage.mp. (0)

55 artificially sweetened.mp. (124)

56 or/1-55 (281239)

57 limit 56 to (english language and humans) (90121)

58 "randomized controlled trial".pt. (389607)

59 (random$ or placebo$ or single blind$ or double blind$ or triple blind$).ti,ab. (826601)

60 (retraction of publication or retracted publication).pt. (6940)

61 or/58-60 (915070)

62 (animals not humans).sh. (3922158)

63 ((comment or editorial or meta-analysis or practice-guideline or review or letter or journal correspondence) not "randomized controlled trial").pt. (3312729)

64 (random sampl$ or random digit$ or random effect$ or random survey or random regression).ti,ab. not "randomized controlled trial".pt. (50474)

65 61 not (62 or 63 or 64) (684058)

66 exp cohort studies/ (1402497)

67 cohort$.tw. (297337)

68 controlled clinical trial.pt. (89898)

69 epidemiologic methods/ (30231)

70 limit 69 to yr=1971-1988 (9464)

71 or/66-68,70 (1609030)

72 65 or 71 (2121434)

73 57 and 72 (13927)

74 taste reaction.mp. (6)

75 cancer.mp. or exp Neoplasms/ (2874302)

76 Orbitofrontal cortex.mp. (3872)

77 gustation vagal.mp. or exp Taste Perception/ (719)

78 exp Enteroendocrine Cells/ or enteroendocrine.mp. or exp Endocrine Glands/ (366841)

79 exp Receptors, G-Protein-Coupled/ or gut taste.mp. (272864)

80 exp Feeding Behavior/ or altered eating behavior.mp. (126201)

81 meal size.mp. (1260)

82 portion size.mp. or exp Portion Size/ (654)

83 serving size.mp. or exp Serving Size/ (171)

84 exp Food Habits/ or dietary pattern.mp. (147041)

85 food preference.mp. or exp Food Preferences/ (10627)

86 food intake.mp. or exp Eating/ (82086)

87 energy intake.mp. or exp Energy Intake/ (43617)

88 exp Intestinal Absorption/ or meal absorption.mp. (40452)

89 night eating syndrome.mp. (171)

90 food addiction.mp. (162)

91 body composition.mp. or exp Body Composition/ (47486)

92 exp Blood Glucose/ or glycemia.mp. (135287)

93 fullness.mp. (3272)

94 exp Satiety Response/ or satiety.mp. or exp Satiation/ (8881)

95 exp Appetite/ or desire to eat.mp. (8594)

96 appetite regulation.mp. or exp Appetite Regulation/ (2958)

97 hunger.mp. or exp Hunger/ (15721)

98 gastric emptying.mp. or exp Gastric Emptying/ (12824)

99 gastrointestinal motility.mp. or exp Gastrointestinal Motility/ (34614)

100 stomach.mp. or exp Stomach/ (235044)

101 limit 100 to yr="1966 - 1977" (36128)

102 or/74-99,101 (3968311)

103 73 and 102 (4440)

***************************
