# Supplementary material for: Creating a literature database of low-calorie sweeteners and health studies: evidence mapping
Source: BMC Med Res Methodol. 2016 Jan 5;16:1. doi: 10.1186/s12874-015-0105-z (PMC4700619; doi:10.1186/s12874-015-0105-z)
Supplement: Additional file 2: — LCS Database Manual and Codebook. (DOCX 56 kb) [file 12874_2015_105_MOESM2_ESM.docx]

**LCS Database Manual and Codebook**

Principal Investigator: Mei Chung, MPH, PhD ([Mei_Chun.Chung@tufts.edu](mailto:Mei_Chun.Chung@tufts.edu))

Data Manager: Ding Ding Wang, MPH ([deenawang@gmail.com](mailto:deenawang@gmail.com))

Tufts University

Supported by North American branch of the International Life Sciences Institutes (ILSI), Carbohydrates Committee. *More information on Low-calorie sweeteners can be found at:* [*http://www.ilsi.org/NorthAmerica/Pages/LowCalorieSweeteners.aspx*](http://www.ilsi.org/NorthAmerica/Pages/LowCalorieSweeteners.aspx)*. ILSI North America is a public, nonprofit foundation that provides a forum to advance understanding of scientific issues related to the nutritional quality and safety of the food supply by sponsoring research programs, educational seminars and workshops, and publications. ILSI North America receives support primarily from its industry membership, which works collaboratively with academic scientific advisors on the development of its programs.*

**Introduction**

Low-calorie sweeteners (LCS), also known as sugar substitutes, non-nutritive sweeteners, or artificial sweeteners, are ingredients added to foods and beverages to provide sweetness without adding a significant amount of calories. A large body of literature evidence exists on low-calorie sweeteners yet the health effects of LCS beyond body weight or composition remain unknown and have not been systematically reviewed. As evidence and interest on LCS continues to grow, there is the need to understand the potential effects of low-calorie sweeteners. LCS evidence map is a database that collects the features and characteristics of the LCS studies. More specifically, it includes data on study design (study design and duration), population (baseline health status, age, sample size, anthropometrics), intervention/comparison (type of LCS, comparisons or control groups, numbers of people analyzed, forms of administration), and outcome information (all outcomes/endpoint reported in the full text). In addition, study aim/hypothesis and funding source were also collected. (See Database Codebook Table). Outcomes were also coded into clinically and biologically relevant outcome groups, generated by Tufts research team and stakeholder panel, to better describe data in the map (See Outcome Groups Table).

The goal of this Low-calorie sweetener evidence map is to provide existing low-calorie sweeteners studies readily in a database, describe the studies, and assist health researches in linking LCS to health outcomes efficiently

**Objectives**

- Provide a database of existing literature on LCS and health outcomes.
- Index key features and characteristics for quick summary report generation.
- Help identify LCS and health effect of interest and identify gaps in LCS research
- Systematically collect information to support further evidence synthesis

**Summary of Methods**

**Scope.** ILSI and Tufts research team determined the original scope of the map, designed search strategy and conducted search on Ovid Medline®

- Energy sensing and Objective sweet tasting
- Appetite and Satiety
- Glycemic and Hormones
- Dietary intake and Energy intake
- Body weight and/or Body composition

**Low-Calorie Sweeteners.** Approved by FDA or Generally regarded as safe (GRAS) by FDA:

- Acesulfame potassium (Acesulfame K, Ace K): Sunett, Sweet One, E950
- Saccharin: Sweet ‘N Low, Sweet Twin, Sugar Twin, Necta sweet, Sodium saccharin, Calcium saccharin, Acid saccharin
- Sucralose: Splenda, Sukrana, SucraPlus, Candys, Cukren, Nevella
- Advantame
- Stevia (Rebaudioside A, Reb-A, rebiana A): sweet leaf, sun crystals, steviva, Truvia, Pure Via
- Swingle fruit (Luo Han Guo, monk fruit): Siraitia grosvenorii
- Sugar alcohols: Lacitol, Erythritol, Sobitol, Xylitol, Mannitol, Maltitol,
- Trehalose, Tagatose

**Selection Criteria**

| **Inclusion** | **Exclusion** |
| --- | --- |
| English | Animal studies |
| Human subjects | In vitro cell studies |
| Interventions studies (randomized or non randomized control trials or single) | Case control, cross-sectional; Reviews, interviews, bibliographies, letters, or guidelines |
| Adults; Pregnant women and infants (>6mo) | Systematic reviews and meta-analysis |
| Prospective cohort studies | Cancer patients |
| FDA-approved sweeteners | Non-oral intake |

**Literature Search and Selection Process.**

**Data Extraction**

- Abstract screening (10% double screening)
- Abstract PICO extraction (Population, Intervention/Exposure, Comparator, Outcome)
- Full-text article screening (double screening)
- Full-text article extraction (More details on study type and design, demographics, interventions, and outcomes)

**Database Codebook**

| **Variable** | **Variable name** | **Description** | **Comments** |
| --- | --- | --- | --- |
| PMID | Pubmed ID |  |  |
| Title | Title |  |  |
| Author | First author |  |  |
| Year | Publication Year |  |  |
| Population | Study population | Numeric value |  |
| Intervention (exposure) | Exposures | Exposures, name of LCS, separated by comma |  |
| Comparator | Comparators | Comparators separated by comma |  |
| Outcomes | Outcomes reported, | Outcomes separated by semi-colon |  |
| LCS vs Sugar | Intervention comparison type | 1 = LCS vs Sugar focusing on LCS  2 = Sugar vs LCS focusing ono sugar |  |
| Study Design | Study design | RCT-c, RCT-p, nRCT-c, nRCT-p, Single arm, unspecified |  |
| Country | Country | Text |  |
| Age | Age | Mean (range) |  |
| % Male | Population male percentage | Percentage |  |
| BMI | BMI | Mean (range), kg/m^2^ |  |
| Intervention Form | Form of the intervention taken | Beverage, food or meal, supplement, oral rinse |  |
| Sample size | Sample size | Numeric |  |
| Number analyzed (control) | Sample size analyzed in control group | Numeric |  |
| Number analyzed (intervention) | Sample size analyzed in intervention group | Numeric |  |
| Baseline health | Baseline health status | **Healthy.** Generally healthy  **Overweight**. Overweight, obese, overweight and/or obese, partially obese  **Diabetes.** Type 2 diabetes, Type 1 diabetes, Type 2 diabetes, Type 1 and Type 2 diabetes.  **Mixed.** Mixed population  **Other**. Hypercholesterolemia, hyperglyceridemia, coronary heart disease, chronic kidney disease, myocardial infarction, non-alcoholic fatty liver disease, hospitalized |  |
| Duration | Study duration | <1 day  2 days-1 month  1-6 months  6 months-1 year  >1 year |  |
| Brain | Alter Energy Sensing Outcome group | 0 = no  1 = yes | Outcomes included |
| Glycemic | Glycemic and Gastric hormone Outcome group | 0 = no  1 = yes | See outcome table for outcomes included |
| Appetite | Appetite and Satiety Outcome group | 0 = no  1 = yes | See outcome table |
| Dietary | Energy intake and Dietary intake Outcome group | 0 = no  1 = yes | See outcome table |
| Bodyweight | Body weight and/or body composition Outcome group | 0 = no  1 = yes | See outcome table for outcomes included |
| Title outcome | Most important outcome | Most important outcome reported by title/abstract |  |
| Hypothesis | Hypothesis/aim | Hypothesis of the paper |  |
| Funding source | Funding source |  |  |
| Funding source (coded) |  | Government;  Industry;  Non Profit (NP);  Government & Industry;  Government & NP;  NP & Industry;  Multiple foundation;  Not reported |  |

*NR = NOT REPORTED

**Outcome Groups**

Outcomes related to appetite/satiety ratings such as hunger score and desire to eat were often rated by a Visual Analog Scale and grouped under the Appetite category. Outcomes focused on neurological measurements and sensing signals by the brain were grouped in the Altered Energy sensing category. Body weight, body composition and changes in weight-related outcomes were grouped under the Body weight/body composition category. The category of Dietary Intake included groups such as energy intake, dietary intake, food intake, and carbohydrate intake. Glucose, insulin, and gastric hormones were grouped under the Glycemic category. We coded outcome groups next to the list of outcomes in the evidence map so that either the full outcome list or the outcome group can be used to output analysis.

| **Outcome Groups** | **Outcomes included** |
| --- | --- |
| **Appetite** | Appetite Ratings using VAS: hunger, desire to eat, fullness, prospective consumption, thirst, motivational and behavioral reported through questionnaire |
| **Altered energy sensing by brain** | Neurological measurements (fMRI, EEG), sensory rating (sweetness, intensity, pleasantness, sensory specific satiation), taste, perception and preference, taste Reaction time |
| **Body weight/**  **body composition** | Body weight, body composition, BMI, waist circumferences, weight/BMI changes |
| **Dietary intake** | Energy Intake, dietary intake, food intake, carbohydrate intake, sugar intake, salt intake, water intake |
| **Glycemic** | Glucose, HbA1c, insulin concentration/sensitivity, hypoglycemia, insulin, glucagon, glucose-dependent insulinotropic peptide (GIP), glucagon-like peptide-1 (GLP-1), peptide tyrosine tyrosine (PYY), cholecystokinin (CCK), enterostatin, ghrelin, leptin, somatostatin, oxyntomodulin |
